# Extrapolation of oritavancin PK/PD targets to inform therapeutic drug monitoring: a systematic review

**DOI:** 10.1093/jac/dkag117

**Published:** 2026-03-31

**Authors:** Giammarco Baiardi, Emanuele Pontali, Valeria Marini, Maria Luisa Cristina, Marina Sartini, Francesca Mattioli

**Affiliations:** Laboratory Medicine Unit, Department of Laboratory Diagnostics, IRCCS Ospedale Policlinico San Martino, Genoa 16132, Italy; Infectious Disease Unit, EO Ospedali Galliera, Genoa 16128, Italy; Pharmacology and Toxicology Unit, Department of Internal Medicine, University of Genoa, Genoa 16132, Italy; Clinical Pharmacology Unit, EO Ospedali Galliera, Genoa 16128, Italy; Department of Health Sciences, University of Genoa, Genoa, Italy; Hospital Hygiene Unit, Ente Ospedaliero Ospedali Galliera, Genoa, Italy; Department of Health Sciences, University of Genoa, Genoa, Italy; Hospital Hygiene Unit, Ente Ospedaliero Ospedali Galliera, Genoa, Italy; Pharmacology and Toxicology Unit, Department of Internal Medicine, University of Genoa, Genoa 16132, Italy; Clinical Pharmacology Unit, EO Ospedali Galliera, Genoa 16128, Italy

## Abstract

**Background and objectives:**

Oritavancin is a lipoglycopeptide with sustained bactericidal activity against Gram-positive bacteria due to its prolonged half-life. This Systematic Review aimed to extrapolate, from *in vitro/in vivo* or clinically study, the most relevant PK/PD target to inform therapeutic drug monitoring-guided oritavancin dose optimization in clinical practice.

**Materials and methods:**

Following the PRISMA 2020 Statement and adopting the PICO strategy, a comprehensive search was conducted in PubMed, Scopus and Cochrane databases up to September 2025.

**Results:**

Of 186 articles screened, 52 were considered eligible for full-text assessment. Nine studies were included and proceeded with data extraction and synthesis steps. *In vitro* studies showed a marked concentration-dependent bactericidal activity at *f*C_max_ > 4–16 mg/L against different bacterial strains, further confirmed by *in vivo* animal models (*f*C_max_/MIC > 6 to 14). However, the only identified in-human daily repeated doses study supported the ﬁndings of an exposure–response relationship with %*fT* > MIC as predictive of microbiological and clinical success.

**Conclusions:**

The peculiar pharmacokinetics profile of oritavancin results in a borderline collinearity between the two PK/PD indices *f*C_max_/MIC and %*f*T > MIC in relation to microbiological and clinical success rates. On the basis of available *in vitro*/*in vivo* data supporting concentration-dependent killing activity, a single 1200 mg oritavancin dose should be adequate for most infections. In special patient populations, or when multidose oritavancin regimens are adopted for long-term antibiotic treatment, therapeutic drug monitoring supported by expert clinical pharmacological advice may be valuable to optimize the initial and next-dose strategy (1200 mg or 800 mg) and to define the timing of re-administration.

## Introduction

Oritavancin is a semi-synthetic lipoglycopeptide antibiotic approved by the FDA in 2014^1^ and EMA in 2015^2^ for the treatment of acute bacterial skin and skin structure infections (ABSSSIs) caused by Gram-positive organisms in both adults and paediatric patients aged 3 months and older.^1,2^

The chemical structure of oritavancin resembles that of vancomycin; however, it is derived from the natural glycopeptide chloroeremomycin through the addition of an *N*-alkyl-p-chlorophenylbenzyl substituent on the disaccharide moiety, which enhances its activity against vancomycin-sensitive and vancomycin-resistant *Enterococci* (VSE and VRE).^3,4^ As mechanism of action shared with all other glycopeptides and lipoglycopeptides, oritavancin inhibits the transglycosylation step of cell-wall synthesis in Gram-positive bacteria. In addition, oritavancin inhibits the transpeptidation step of cell-wall synthesis by binding to the bridging segment of the cell-wall peptidoglycan, a secondary binding site that has not been demonstrated for vancomycin.^5–7^ This secondary mechanism leads to the loss of the cell-wall integrity in Gram-positive bacteria, causing depolarization, permeabilization and rapid cell death.^8,9^

The pharmacokinetics of oritavancin are most commonly described by a three-compartment model, with a rapid initial distribution phase followed by a slower and longer elimination phase and a very prolonged terminal half-life (245–393 h).^10,11^ The drug exhibits extensive tissue distribution properties (volume of distribution ∼87.6 L), confirmed by diffusion in to blister fluids^12^ and accumulation within lysosomes of several eukaryotic cells.^13,14^ Oritavancin is highly bound to human plasma proteins (∼85%–90%).^15^ The drug is apparently not metabolized and is eliminated slowly from tissue sites with <5% recovered in the urine and 1% in the faeces over 7 days after dosing.^16^

The approved dose of oritavancin for adults with ABSSSI is 1200 mg administered as a single intravenous infusion (IV) over 3 hours^17^ or over 1 hour using the newer improved formulation.^18,19^

Pooled safety data from the SOLO studies^20^ indicate that aa single 1200 mg dose is generally well tolerated, with a safety profile similar to that of vancomycin.

The long half-life of oritavancin compared with vancomycin, did not result in a clinically meaningful delay in the onset or prolongation of adverse events. However, clinical caution should be exercised when oritavancin is employed in multidose regimens, as exposure-related toxicity may arise due to PK alterations during prolonged treatment or in frail and/or special patient populations.

Indeed, due to its long half-life and broad Gram-positive activity, clinical interest in the use of repeated doses of oritavancin is arising for the treatment of complicated infections such as bacteraemia, endocarditis, pneumonia, osteomyelitis and surgical site infections.^21^ However, there is still limited consensus regarding the optimal number of administrations, dosage and dose frequency when oritavancin is used in multidose regimes.^22,23^

The efficacy of antimicrobial therapies generally depends on the attainment of pharmacokinetic/pharmacodynamic (PK/PD) indices that correlate with the microbiological and/or clinical success rate.^24–26^

Key PK/PD indices include the maximum concentration to MIC ratio (*C*_max_/MIC), the 24 h area under the curve to the MIC ratio (AUC/MIC), and the percentage of time during which drug concentrations are above the MIC (%T > MIC). These parameters vary among antimicrobial classes and serve as clinical indicators of therapeutic efficacy, which is driven by the free (unbound) drug concentration responsible for pharmacological activity. However, consensus regarding the PK/PD index that best predicts oritavancin clinical efficacy in single- or multidose regimens is still lacking. For highly protein-bound agents such as oritavancin (∼85%-90% bound), analysis of the relationship between free drug exposure and pharmacological response may better explain clinical efficacy. This is particularly relevant for drugs approved with fixed dosing regimen (such as oritavancin), where total drug exposure may show limited interindividual variability, while differences in the pharmacologically active free fraction may influence therapeutic response.

Although free drug concentrations are not routinely measured in clinical practice, analysis of oritavancin levels may provide valuable insights into its pharmacological activity. In this context, therapeutic drug monitoring (TDM) represents an important tool for translating PK principles into clinical decision-making.

TDM is indeed a widely used tool in clinical practice to define the optimal timing of re-administration of other lipoglycopeptides.^27,28^ However, a clearly defined PK/PD target to aid TDM-guided expert clinical pharmacological advice for oritavancin remains debated.

The identification of clinical PK/PD targets derived from non-clinical *in vivo/in vitro* infection models represent a fundamental step in advancing the understanding of antimicrobial pharmacology.

The objective of this Systematic Review was to extrapolate, from available *in vitro/in vivo* or clinically study, the most relevant PK/PD target to inform oritavancin TDM-guided dose optimization supported by expert clinical pharmacological advice in routine clinical practice.

## Materials and methods

This systematic review of the literature was registered in the PROSPERO database (CRD42024612220) and conducted in accordance with the Preferred Reporting Items for Systematic Reviews (PRISMA 2020) Statement^29^ to ensure the adherence to current reporting standards.

A comprehensive search on the PubMed, Scopus and Cochrane databases was performed. The search string used to identify eligible studies across the three databases, from inception to September 2025 was based on a combination of Medical Subject Headings (MeSH) terms as vocabulary and, where appropriate, a wild-card option. The research question was structured to ensure informative study retrieval and to prevent unnecessary searches using the PICO strategy.

The comprehensive search strategy adopted is shown in Table 1. Briefly, the inclusion criteria were: (i) preclinical *in vitro/in vivo* PK/PD studies on oritavancin, clinical studies, observational studies and case report/series including TDM of oritavancin concentrations and (ii) PK/PD analysis, population PK/PD analysis and target attainment simulations investigating PK/PD relationships predictive of oritavancin efficacy. The exclusion criteria were: (i) articles not strictly related to the research query; (ii) papers without sufficient information on dosage and (iii) research work not matching the PICOS criteria. No filters of time or language were applied. For further details of the search strategy, see Table 1.

**Table 1.** Search strategy adopted in the present systematic review

Search strategy	Details	
Search string	(‘oritavancin’[Title/Abstract]) AND (‘Pharmacokinetics’[MeSH Terms] OR ‘Drug monitoring’[MeSH Terms] OR ‘pharmaco*’[Title/Abstract] OR ‘PK’[Title/Abstract] OR ‘PD’[Title/Abstract] OR ‘PK/PD’[Title/Abstract] OR ‘model*’[Title/Abstract] OR ‘TDM’[Title/Abstract] OR ‘therapeutic drug monitoring’[Title/Abstract])	
Inclusion criteria	P (patients/population):	Patients/animals treated with oritavancin-based regimens, or oritavancin *in vitro*/*in silico* studies
	I (intervention/exposure):	Oritavancin-based regimens therapeutic administration with drug monitoring or oritavancin PK/PD analysis
	C (comparisons/comparators):	no comparator
	O (outcome):	Oritavancin PK/PD target for TDM
	S (study design):	Preclinical studies, Clinical studies, observational studies, case report/series, in silico studies
Databases	PubMed, Scopus and Cochrane	
Exclusion criteria	Study design: articles with insufficient PK/PD details	
Time filter	None (from inception)	

After removal of duplicates by M.S., articles were independently screened by two authors (G.B. and F.M.) on the basis of title and abstract using the Rayyan platform for Systematic Review.^30^ Any case of disagreement was solved by discussion until consensus was reached or if discordance persisted, a third researcher (E.P.) was consulted.

The full texts of relevant identified studies were then further acquired and assessed against the inclusion/exclusion criteria by four authors (G.B., E.P., V.M. and F.M.). Each reference for the selected articles was then checked to not to miss any relevant studies.

Data extraction and synthesis of the included articles were carried out by three authors (G.B., M.L.C. and M.S.) to determine type of study, PK/PD parameters investigated, which dosing regimen was adopted and the most relevant PK/PD index for TDM-guided efficacy target attainment resulting from data synthesis.

## Results

**Figure 1.**
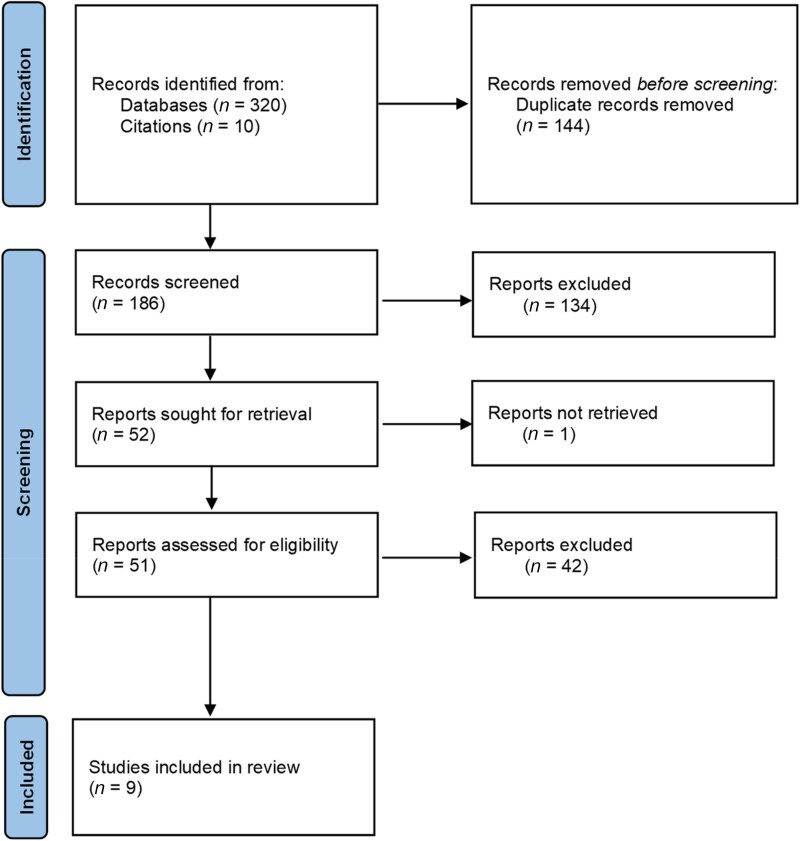
Systematic review Prisma 2020 flow-chart.

The search query yielded 140 articles in PubMed, 158 in Scopus and 22 in Cochrane, resulting in a total of 330 articles hits after the inclusion of 10 articles by citation retrieval. After removal of 144 duplicates, 186 were screened in Rayyan on the basis of their title and abstract. During this procedure, 134 articles were excluded due to irrelevance. The remaining 52 articles were considered eligible for full-text assessment, which was conducted on 51 articles after the exclusion of one record that could not be retrieved. The data extracted from the nine articles included at the end of the Systematic Review process (Figure 1) are presented in Table 2.

**Table 2.** Summary of findings from the included studies

	Article	Study design	PK/PD parameters investigated	Oritavancin dosage regimen	Key points	Relevant PK/PD index for TDM^a^
1	Mercier *et al.* 1997^31^	*in vitro*	Time killing kinetics	ND	Oritavancin exhibited bactericidal activity against a strain of MRSA at concentration 4 × MIC Furthermore, Oritavancin exhibited concentration-dependent killing effect against VREF	C > 4xMIC
2	Boylan *et al.* 2003^32^	*in vivo*	*fC*_max_/MIC *f*AUC/MIC *fT* > MIC	Single IV^b^ dose escalation 0.25–20 mg/kg	With Inhibitory Emax model, Oritavancin *C*_max_ (*r*^2^ 0.93) appears to correlate better with bactericidal activity than do *T* > MIC (*r*^2^ 0.84) and AUC (*r*^2^ 0.54) Bacterial stasis as associated with a *fC*_max_/MIC ratio of ∼6 and %*fT* > MIC of ∼17% to 20%. Whereas, near-maximal effect was associated with a *fC*_max_/MIC ratio of ∼14 and %*fT* > MIC of ∼42% to 50% These data suggest that Oritavancin efficacy appears better optimized by high *C*_max_ rather than by prolonged unbound concentrations above MIC	*fC*_max_/MIC > 6 and %*fT* > MIC of ∼17% to 20% for bacterial stasis *fC*_max_/MIC > 14 and %*fT* > MIC of ∼42% to 50% for maximal effect
3	Bhavnani *et al.* 2006^33^	PK/PD in-human	*fC*_max_/MIC *f*AUC_0-24_/MIC *%f*T > MIC	Four IV^c^ dose escalation 5–10 mg/kg/day	The evaluation of PK/PD relationships for efficacy among patients with bacteraemia has been limited The probability of microbiological success was 93% for those patients with a %*f*T > MIC ≥22% The probability of clinical success was 87% for those patients with a %*f*T > MIC ≥22% Logistic regression analysis revealed a relationship between microbiological response and %*f*T > MIC (OR = 4.42, *P* = 0.09)	*%f*T > MIC ≥22%
4	McKay *et al.* 2009^34^	*in vitro*	*fC*_max_ *fC*_trough_ Time killing kinetics	4 mg/L (*fC*_max_ from 200 mg) 0.5 mg/L (*fC*_trough_ from 200 mg) 16 mg/L (*fC*_max_ from 800 mg) 2 mg/L (*fC*_trough_ from 800 mg)	Oritavancin displayed concentration-dependent killing of MSSA, MRSA, VRSA, VISA, VSE and VRE strains At predicted *fC*_max_ from a 200 mg dose in humans (4 mg/L), oritavancin exerted bactericidal activity (−3 log_10_ kill) against MSSA, MRSA and VRSA within 1 h and against VSE between 11 and 24 h At predicted *fC*_max_ from an 800 mg dose (16 mg/L), oritavancin was bactericidal against VISA strains at 24 h and against VRE at 10 h	*fC*_max_ > 4 mg/L for bactericidal effect against MSSA, MRSA, VRSA, VSE *fC*_max_ > 16 mg/L for bactericidal effect against VISA and VRE
5	Belley *et al.* 2009^35^	*in vitro*	*fC*_max_ *fC*_trough_ Time killing kinetics	4 mg/L (*fC*_max_ from 200 mg) 0.5 mg/L (*fC*_trough_ from 200 mg) 16 mg/L (*fC*_max_ from 800 mg)	Oritavancin exhibited concentration-dependent bactericidal activity against stationary-phase MSSA, MRSA and VRSA strains at its *fC*_max_ 200 and *fC*_max_ 800	*fC*_max_ > 4–16 mg/L for bactericidal effect against stationary-phase MSSA, MRSA and VRSA
6	Ambrose *et al.* 2012^36^	*in vivo*	*fC*_max_/MIC *f*AUC/MIC *fT* > MIC	20 mg/kg	*C*_max_ best predicted oritavancin activity (*r*^2^ = 96%), followed by *T* > MIC (*r*^2^ = 83%) and AUC (*r*^2^ = 77%) on a log scale	*fC*_max_/MIC
7	Rose, W. E., & Hutson, P. R. (2020)^37^	*in silico*	*T* > MIC AUC > MIC	Two-dose regimen: 1200 mg at day 0 + 800 mg at day 7	Oritavancin two-dose regimen grants concentrations >0.12 mg/L (susceptibility breakpoint) for 8 weeks (simulation for total drug) and 4.6 weeks (unbound free drug) Oritavancin two-dose regimen grants AUC/MIC simulation for total drug ≥17.568^d^ (free unbound >2.635.2) for MIC ≤ 0.25 mg/L	ND
8	Bongiovanni *et al.* (2024)^38^	TDM Case Series	*fC*_trough_ > MIC	Case 1: 1200 mg, then 800 mg q10d × 4 Case 2: 1200 mg, q10d × 5	TDM is an essential tool to individualize and tailor Oritavancin dosing to achieve appropriate concentration–time profile and AUC/MIC in each patient	*fC*_trough_ > MIC
9	Buonomo *et al.* (2024)^39^	TDM Case Report	AUC/MIC > 17.568^d^	10 outpatients sequential 1200 mg doses over 28 weeks, TDM-guided	Oritavancin may be an effective option for long-term suppressive therapy Further studies are needed to define PK targets and optimal schedule of administration	ND

^a^Most relevant PK/PD index identified for oritavancin TDM.

^b^Single IV oritavancin dose escalation 0.25–20-mg/kg.

^c^Patients were randomly assigned to 10–14 days of oritavancin or control; four sequential IV oritavancin regimens (5, 6.5, 8 or 10 mg/kg/day) were infused over 60–90 min (10 mg/kg/h).

^d^AUC/MIC > 17.568 is cited over different works as the preliminary PK/PD target identified during early clinical studies for oritavancin clinical response in patients, which was presented by Bhavnani *et al.* (2014) in a poster at the 54th Interscience Conference on Antimicrobial Agents and Chemotherapy (Washington, DC, USA). 5–9 September 2014; however, its full text is not retrievable from the literature (Bhavnani SM, Hammel JP, Rubino CM, *et al.* Oritavancin pharmacokinetic-pharmacodynamic analyses for efficacy based on data from patients with ABSSSIs enrolled in SOLO I and II. Poster A-1309. 54th Interscience Conference on Antimicrobial Agents and Chemotherapy (Washington, DC, USA). 5–9 September 2014).

*C* > 4×MIC: drug plasma concentration exceeds four times the MIC; *f*AUC_0–24_/MIC: ratio of free 24-hour area under the concentration–time curve to the MIC; *f*C_trough_ > MIC: free trough plasma concentration above the MIC; ND, not determined.

The included studies spanned a period of 27 years from 1993 to 2024.

The studies from Mercier *et al.*,^31^ McKay *et al.*^34^ and Belley *et al.*^35^ reported *in vitro* time-kill studies using different bacterial strains, whereas Boylan *et al.*^32^ and Ambrose *et al.*^36^ reported *in vivo* results from neutropenic mouse models of infection.

Bhavnani *et al.*^33^ was the only identifiable in-human efficacy PK/PD study.

Rose and Hutson^37^ attempted to estimate the efficacy of a two-dose oritavancin regimen through *in silico* trial simulation analysis, while Bongiovanni *et al.*^38^ and Buonomo *et al.*^39^ reported a case series/report on TDM-guided multidose oritavancin.

Given the heterogeneity of study designs (*in vitro/in vivo* experiments, simulations and case reports), formal risk-of-bias assessment tools such as the Cochrane RoB or ROBINS-I were not considered applicable.

## Discussion

The prolonged plasma half-life of oritavancin (245–393 h) makes it challenging to distinguish the effects of the key PK parameters *C*_max_, AUC and %T > MIC on microbiological and/or clinical success rate following single or multidose oritavancin treatment.

Analysis of the included *in vitro* microbiological efficacy studies (Table 2, study numbers 1, 4 and 5), suggests that *fC*_max_ appears to have the best correlation with bactericidal activity against different bacteria strains at concentrations mimicking clinical *fC*_max_ drug plasma level deriving from single dose of 200 mg (>4 mg/L) to 800 mg (>16 mg/L). In particular, *fC*_max_ > 4 mg/L appears to be bactericidal against MSSA, MRSA, VRSA and VSE strains, whereas *fC*_max_ > 16 mg/L appears to be required for bactericidal effect against VISA and VRE strains.^34^ Interestingly, at *fC*_max_ > 4–16 mg/L oritavancin also exhibits concentration-dependent bactericidal activity against stationary-phase MSSA, MRSA and VRSA strains,^35^ suggesting potential activity against biofilm-forming colonies in difficult-to-treat sites of infection, such as prosthetic joint infection, vascular catheters, pacemakers or in the case of endocarditis (native or prosthetic valves).

*In vivo* animal models (Table 2, study numbers 2 and 6) confirmed the marked concentration-dependent killing observed *in vitro,* as *fC*_max_/MIC (ratio of peak free drug plasma concentration to the MIC) was identified as the PK/PD index with the strongest association with efficacy.^32^ In particular, Boylan *et al. 2003* (*in vivo* mice model of *Staphylococcus aureus* infection) reported bacterial stasis as associated with a *fC*_max_/MIC ratio of ∼6 and a percentage value of time the free drug plasma concentration remains above the MIC (%*f*T > MIC) of ∼17% to 20%. Whereas near-maximal was reported with an *f*C_max_/MIC ratio of ∼14 and a %*f*T > MIC of ∼ 42% to 50%.^32,33^

Although *C*_max_ (*r*^2^, 0.96) appeared to correlate slightly better with bactericidal activity *in vivo* than %T > MIC (*r*^2^ 0.83), this borderline collinearity between the two PK/PD measures is not unexpected, since the long half-life of oritavancin grants concentrations above the MIC for 100% of time in most cases.^32,36^ Indeed, a single oritavancin dose of 1200 mg is expected to maintain free drug concentration above the *Staphylococcus aureus* EUCAST clinical breakpoint of 0.12 mg/L for a median duration of ∼21 days^37^ in most patients.

Consequently, the only identified in-human study^33^ (Table 2, study number 3) supported the ﬁndings of an exposure–response relationship between %*f*T > MIC and microbiological response, with a similar association observed for clinical response.

The probability of microbiological success was 93% and the probability of clinical success was 87% in patients with a %*f*T > MIC ≥22%.^33^ However, this proposed PK/PD target should be carefully interpreted, as it was derived from a context of repeated low daily dosing regimen, whereas nowadays oritavancin is approved as a single 1200 mg infusion or is prescribed off-label as multidose regimens of 1200 mg or 800 mg.

The high microbiological and clinical success rates in-human^33^ observed at a relatively low %*f*T > MIC threshold (≥22%) may be explained by the peculiar PK/PD properties of oritavancin. These include: a marked initial concentration-dependent killing effect mediated by permeabilization of the bacterial wall causing rapid cell death induced by its secondary mechanism of action,^8,9^ which is then further sustained over time by its primary mechanism of action of cell-wall synthesis disruption.^3,4^ Moreover, the very long elimination half-life (245–393 h) and accumulation within macrophages^13,14^ may further facilitate drug delivery to difficult-to-access infection sites.

Although the only included in-human PK/PD study involved a relatively large sample size for the peculiarity of the treated infection type (bacteriemia) and drug investigated,^33^ the available clinical evidence remains insufficient to draw definitive conclusions regarding the role of TDM for oritavancin in routine practice. Similarly, the single *in silico* study (Table 2, study number 7) and the two identified TDM case report/series (Table 2, study numbers 8 and 9) do not provide robust evidence to establish validated exposure targets.

Nevertheless, based on available *in vitro*/*in vivo* data of concentration-dependent killing of MSSA, MRSA, VRSA, VISA, VSE and VRE strains at *fC*_max_ > 4–16 mg/L (Table 2), and considering that the new improved oritavancin formulation provides a mean *C*_max_ of ∼148 mg/L following a 1-hour infusion,^18,19^ it can be hypothesised that a single 1200 mg dose should be sufficient to treat most infection types in most patients cases. This assumption is supported by the drug’s prolonged half-life, which ensures sustained %*f*T > MIC, along with an estimated free peak concentration (*f*Cmax) of ∼14.8–22.2 mg/L, values consistent with bactericidal thresholds identified in preclinical models when assuming protein binding ranging from 85% to 90%^15^.

However, in special patient populations with potential PK alterations due to comorbidities (e.g. obesity, impaired renal function, chronic or acute liver failure, etc.) or when multidose regimens are adopted for long-term antibiotic treatment, TDM supported by expert clinical pharmacological advice may be valuable:

- to decide the initial dosing strategy (1200 or 800 mg) based on expected PK variability, aiming to achieve efficacious *fC*_max_ levels without exposing the patient to unnecessarily high concentration levels that may increase the risk of adverse events;
- to determine the best next-dose timing of reinfusion and appropriate dose selection (1200 or 800 mg) to prevent *fC*_min_ values—used as a surrogate of %*f*T > MIC—falling below the EUCAST MIC susceptibility breakpoint of 0.125 mg/L.

### Conclusions

The peculiar pharmacokinetics and multiple mechanisms of action of oritavancin result in a borderline collinearity of the two PK/PD indices *f*C_max_/MIC and %*f*T > MIC in predicting both microbiological and clinical success rate. This raises the possibility that different PK/PD drivers may predominate at different phases of the exposure profile.

On the basis of the available *in vitro*/*in vivo* data of concentration-dependent killing of most bacterial strains at *fC*_max_ > 4–16 mg/L, a single 1200 mg oritavancin dose appears sufficient to treat most infection types in most patients.

When MIC testing is available, it is advisable to verify that the *fC*_max_/MIC ratio is >6 to achieve bacterial stasis or >14 to obtain a near-maximal bactericidal effect.

Considering that the *fC*_max_ resulting from a 1200 mg dose (1-hour infusion time) is ∼14.8–22.2 mg/L in most patients, the *fC*_max_/MIC ratio would be expected to far exceed 14 for strains with MIC ≤ 1 mg/L, ensuring bactericidal activity. However, from an Antimicrobial Stewardship perspective, oritavancin may potentially be overdosed in special patient populations leading to potential adverse effects related to excessive exposure, which should therefore be carefully evaluated.

Indeed, in patients with potentially altered pharmacokinetics or when adopting multidose regimens for long-term therapy, TDM supported by expert clinical pharmacological advice may be most valuable to select the best personalized initial dosing strategy (1200 or 800 mg) to achieve targeted *fC*_max_ > 4–16 mg/L without exposing the patient to the risk of overexposure. It may also support optimization of the next-dose timing of reinfusion and dose selection (1200 or 800 mg) to maintain *fC*_min_ values above the threshold of 0.125 mg/L (*Staphylococcus aureus* EUCAST MIC clinical breakpoint for susceptibility).
